# Effectiveness of the T‐Control catheter: A study protocol

**DOI:** 10.1002/bco2.285

**Published:** 2023-12-04

**Authors:** Yolanda Ramallo‐Fariña, Ana Toledo Chávarri, Adrián Amador Robayna, Max Mòdol Vidal, Cristina Valcárcel‐Nazco, Clara Armas Moreno, Lilisbeth Perestelo‐Pérez, Marta Serrano Muñoz, Manuel Luque González, Lidia García‐Pérez, Miguel Ángel García‐Bello, Pedro Serrano‐Aguilar, Pedro Raúl Castellano Santana, Laura Vera Álamo

**Affiliations:** ^1^ Evaluation Unit (SESCS), Canary Island Health Service Canary Islands Health Research Institute Foundation (FIISC) Tenerife Spain; ^2^ Network for Research on Chronicity, Primary Care, and Health Promotion (RICAPPS) Tenerife Spain; ^3^ Department of Urology University Hospital of Nuestra Señora de Candelaria Tenerife Spain; ^4^ Rethink Medical SL Gran Canaria Spain; ^5^ University of La Laguna (ULL) Tenerife Spain; ^6^ Department of Urology Insular University Hospital of Gran Canaria Gran Canaria Spain

**Keywords:** acute urine retention, catheterisation, cost‐effectiveness, Foley, quality of life, study protocol, T‐Control, urinary tract infection

## Abstract

**Background:**

Foley catheters have been subject to limited development in the last few decades. They fulfil their basic function of draining urine from the bladder but cause other associated problems. T‐Control is a new silicone Foley catheter with an integrated fluid control valve whose design aims to reduce the risks associated with bladder catheterisation by a multifactorial approach. The general purpose of this study is to evaluate the effectiveness and cost‐effectiveness of the T‐Control catheter versus the Foley‐type catheter in patients with Acute Urine Retention (AUR).

**Study design:**

This is a pragmatic, open, multicentre, controlled clinical trial with random allocation to the T‐Control catheter or a conventional Foley‐type catheter in patients with AUR.

**Endpoints:**

The magnitude of infections will be analysed as a primary endpoint. While as secondary endpoint, the following will be analysed: rate of symptomatic and asymptomatic infections; days free of infection; quality of life‐related to self‐perceived health; indication of associated antibiotic treatments; determination of biofilm; number of catheter‐related adverse events; use of each type of catheterisation's healthcare resources; level of satisfaction and workload of health professionals and acceptability of the T‐Control device as well as the patient experience.

**Patients and methods:**

Eligible patients are male adults aged ≥50 years, with AUR and with an indication of bladder catheterisation for at least 2 weeks. The estimated sample size is 50 patients. Patient follow‐up includes both the time of catheter insertion and its removal or change 2 weeks later, plus 2 weeks after this time when the patient will be called for an in‐depth interview.

## 1 BACKGROUND

Urinary bladder catheterisation is a common healthcare procedure for therapeutic and diagnostic purposes,^1^ usually managed by nursing staff.^2^ Overall, 15.5% and 23.6% of hospitalised patients in Europe and the United States, respectively, received an indwelling bladder catheter, with higher rates for older patients, surgery departments and intensive care units (45–79%).^3^

Urethral catheterisation is the most common bladder catheterisation in clinical practice.^4^ Although the available clinical evidence promotes intermittent catheterisation as the first therapeutic option to preserve quality of life^5^, ^6^ and reduce long‐term complication risks,^7^, ^8^, ^9^, ^10^ frequently indwelling catheterisation is required to avoid an additional burden for patients.^11^ In addition to the negative emotions of living with an indwelling catheter,^12^ complications such as urinary tract infections (UTIs), catheter obstruction and recurrent bladder stones^13^, ^14^ might occur. Catheter‐associated urinary tract infections (CAUTI) are common, causing 80% of hospital‐acquired urinary infections,^8^, ^9^ increasing morbidity, mortality^15^ and costs.^16^, ^17^ Moreover, the little scientific literature on the quality of life of patients living with urinary catheters emphasises findings such as lack of autonomy, fear and anxiety and self‐image concerns.^18^

Despite their wide use, limited improvements have occurred in the design and development of indwelling urinary catheters over the last few decades. It has been suggested, however, that a patient‐managed valve connected to the catheter outlet, instead of the conventional urine drainage bag, may lead to improvements,^19^ which enable voluntary and timely opening and bladder emptying by the patient and helping maintain bladder tone and capacity.^20^ This innovation could reduce bladder irritation since periodic filling would reduce contact with the catheter tip. Periodic valve‐regulated flushing might also decrease infection rates and valve blockage.^21^, ^22^, ^23^

T‐Control is a new silicone Foley catheter with an integrated valve to voluntarily control urine flow, which enables bladder filling and its conscious regulation and emptiness, thereby reducing mucosa irritation by contact of the catheter tip. This innovative valve controls urine flow by means of three different positions, from the proximal end of the tube. The ‘open’ and ‘closed’ positions regulate urine flow without other additional accessories required by the conventional Foley catheter. The third or ‘insertion’ position, only available initially for the insertion manoeuvre, prevents unwanted urine leakage thanks to a specific built‐in membrane. T‐Control has an additional safety lock to prevent accidental opening of the catheter valve.

T‐Control has undergone and passed different biocompatibility studies, including the assessment of cytotoxicity, skin sensitisation, intracutaneous reactivity (irritation) and acute, subacute and subchronic systemic toxicity (necessary results to obtain the CE marking). Beyond these pre‐clinical biocompatibility studies, usability studies have also been performed to test the ease of professional handling of T‐Control. These confirm that an easier and safer insertion technique could be provided, while also being able to be performed by one person, which reduces the staffing needs currently recommended by clinical practice guidelines.^24^ Furthermore, patients experienced in the use of bladder catheters, comparatively evaluated T‐Control with conventional catheters and recognised the added value and the intuitive and ease of use of T‐Control.^25^ Finally, in in vitro studies have been observed how T‐Control significantly prevented/delayed the formation and growth of biofilm during the first 5 days, compared with conventional Foley‐type catheter.^26^

This clinical trial protocol shares the methodology to make progress in regard to the process of generating evidence on the effectiveness, safety and cost‐effectiveness of T‐Control, to improve clinical and self‐perceived health outcomes by patients, compared to conventional catheters currently used. In addition, this trial aims to generate information on the potential contribution of T‐Control to improve the sustainability and solvency of the healthcare system.

## 2 STUDY DESIGN

This trial is an open, pragmatic, multicentre, controlled clinical trial with random allocation to T‐Control catheter or traditional Foley catheter.

### 2.1 Trial design and trial setting

The sample will be recruited in the casualty department of two centres in the Canary Islands, Spain: the Hospital Universitario Nuestra Señora de Candelaria (HUNSC, Tenerife) and the Complejo Hospitalario Universitario Insular Materno‐Infantil de Gran Canaria (CHUIMI, Gran Canaria). In addition, patients identified from casualty departments of the healthcare centres associated with both hospitals will also be referred.

### 2.2 Recruitment

Potential participants will be identified by the team of healthcare professionals of the casualty department according to the study's inclusion and exclusion criteria. If the patient meets the criteria, the casualty department team will be able to refer patients directly to the urology department, where the investigator or research assistant will verify the inclusion and exclusion criteria, invite the patient or their relative/caregiver (if necessary) to take part in the study and will request their consent agreement, including the subject on the study's registration sheet.

### 2.3 Random assignment

Participants will be assigned 1:1 to one of the two trial arms by a local research team member using a centralised computerised randomisation system (RAND2 software, The MathWorks Inc, Natick, United States, administered by the data management team, depending on the contract research organisation [CRO]). In order to ensure that the groups are homogeneously distributed in both study centres, a list will be added to the automatic online randomisation system in blocks of four.

The blinded allocation sequence is concealed by the use of a centralised computerised randomisation system. The personnel responsible for catheter insertion will enrol subjects on the randomisation system. The centralised computerised randomisation system will generate the blinded allocation sequence and assign the trial arm.

### 2.4 Blinding

It will not be possible to blind the study arm for the trial subjects, the health professionals, the research team involved and the monitoring team. Data analysis will be blinded to the intervention arm as well as the laboratories that will analyse the urine and catheter samples.

In the event of adverse events that may compromise the patient's safety, data may be unmasked by means of the unique code given to each participant.

## 3 ENDPOINTS

The general purpose of this randomised controlled trial is to evaluate the preliminary effectiveness and cost‐effectiveness of the T‐Control catheter versus the Foley‐type catheter in patients with acute urine retention (AUR).

- Primary endpoint:
- To assess the preliminary effectiveness of T‐Control versus a common Foley catheter, by comparing the magnitude of infections due to the catheter among AUR patients catheterised with the T‐Control device and AUR patients catheterised with the Foley catheter.
- Secondary endpoints
- To assess the preliminary effectiveness of T‐Control versus a common Foley catheter, by comparing the rate of infections (both symptomatic and asymptomatic) due to the catheter among AUR patients catheterised with the T‐Control device and AUR patients catheterised with the Foley catheter. Additionally, evaluate the effectiveness of T‐Control by comparing the days free of infection on the 14th day after catheterisation.
- To assess the preliminary effectiveness of T‐Control versus conventional Foley catheter comparing levels of self‐perceived health‐related quality of life (HRQoL) in AUR patients.
- To compare the indication of antibiotic treatments associated with catheter use between the conventional Foley‐type catheter and the T‐Control device.
- To determine the biofilm and identify the microorganisms that form this to evaluate its relationship with the onset of symptomatic and asymptomatic infections as well as different adverse events.
- To compare the number and relevance of adverse events related to bladder catheterisation between T‐Control and the traditional Foley catheter.
- To assess the preliminary cost‐effectiveness of T‐Control versus the traditional Foley catheter from the public healthcare services perspective.
- To measure and compare the level of satisfaction of health professionals with both types of catheterisation by means of a questionnaire at the end of the study.
- To analyse, by means of qualitative techniques, the acceptability of the T‐Control device and the Foley catheter as well as the patient experience framed within the disease clinical course, identifying the preferences and needs for training and information for use of the device and possible future improvements for the T‐Control device.

## 4 ELIGIBILITY CRITERIA

Patient inclusion criteria are as follows: (1) males with AUR; (2) aged over or equal to 50; (3) absence of UTI symptoms; (4) not having been previously catheterised on the day of inclusion; (5) indication of bladder catheterisation for 2 weeks; (6) maintained cognitive and physical capacity to self‐monitor the catheter valve and (7) signed consent agreement.

Patient exclusion criteria are as follows: (1) current or recent UTI in the last 2 weeks; (2) use of current treatment/antibiotic in the last 2 weeks; (3) immunocompromised patients (diagnosed with advanced cancer, AIDS, etc.); (4) catheter insertion requiring more than one attempt; (5) overactive bladder and (6) patients with bilateral obstructive uropathy.

## 5 METHODS

### 5.1 Interventions

#### 5.1.1 Intervention arm: T‐Control catheter

The T‐control catheter is a flexible, silicone tube with an inflatable balloon at the distal tip, a PTFE membrane integrated into its body and a sliding fluid control valve. The valve, built into the catheter, provides additional functions to the catheter, such as turning urine flow on and off after insertion (functions currently provided by accessories such as caps or valves) and controlling urination during the insertion process (function not provided by any other device). Accidental loss of urine can thus be avoided from the first moment of use until its withdrawal. In addition, it has a safety cap that reduces the possibility of accidental valve movements before use or during transport and fixing. The design has been developed in such a way that, once inserted, it facilitates autonomous use even for the elderly or patients with limited manual dexterity. The device is sterile and single use, like any conventional Foley catheter.

The continuous use of a single device of T‐Control cannot exceed 30 days. The cumulative use for each type of T‐Control device can exceed 30 days.

T‐Control is manufactured, sterilised and packaged by the subcontracted company Conod Medical Co., Ltd under the specifications of Rethink Medical and under its quality standards, as well as its accessory Holder, also manufactured and packaged by the same subcontracted company.

#### 5.1.2 Control arm: Foley catheter

Silicone Foley catheters are transurethral balloon catheters used to treat bladder emptying disorders, as well as drain urine from the urinary tract, continuous fluid irrigation and/or medication administration. It is suitable to be used for a prolonged period of no more than 29 days and in Urology, Internal Medicine, Surgery, Obstetrics and Gynaecology Services.

For this arm, conventional two‐way silicone Foley‐type catheters will be used. They consist of a body, drainage funnel, inflation funnel and balloon valve. The product is sterile and single use.

### 5.2 Study procedures

**FIGURE 1:**
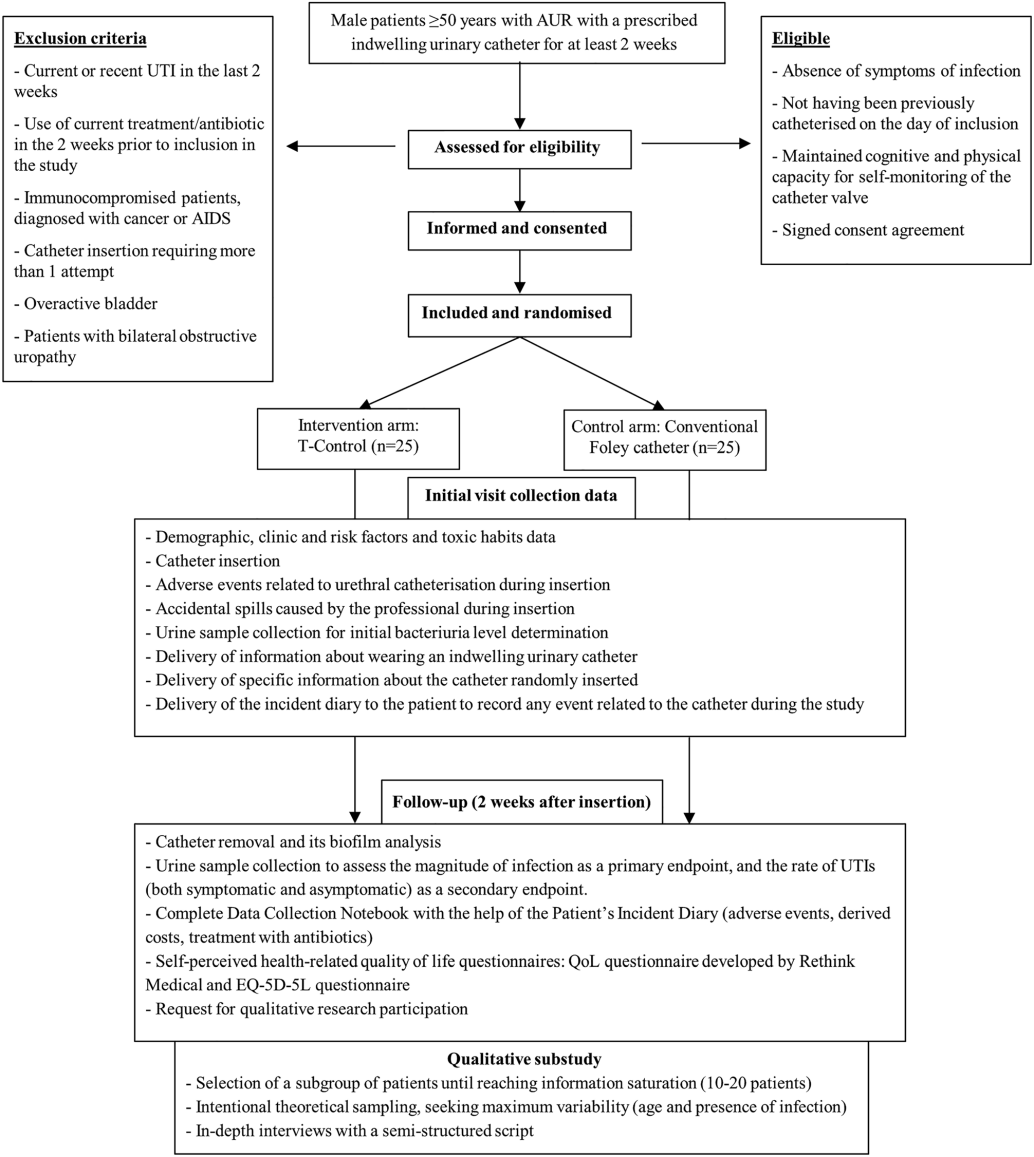
Flow diagram describing the participant timeline through the clinical trial. Abbreviations: AIDS, Acquired Immunodeficiency Syndrome; EQ‐5D‐5L, EuroQol questionnaire – 5 dimensions – 5 levels; QoL, quality of life; UTI, urinary tract infections.

The flowchart in Figure 1 describes the participant timeline throughout the study. The catheter will be inserted by the research staff after obtaining informed consent, and the inclusion and randomisation of the participants. The healthcare professional who inserts the catheter must have experience in bladder catheterisation. Professionals without sufficient experience in catheterisation involved in the study will receive specific training prior to the start of the study. In addition, the research staff will receive specific training on the device prior to the start of the study. All the staff involved in the study will have access to the user instructions at any time.

After catheter insertion, participants and their family, friends or other informal caregivers will receive information about wearing an indwelling urinary catheter and specific information about the device randomly inserted, the standard Foley or the T‐Control catheter. Standard catheter care is permitted during the trial both managed by the participants or participants' caregivers. In addition, patients will receive an incident diary in which they can record any incident during the bladder catheterisation period. Two weeks after indwelling bladder catheter insertion, patients will be contacted by the urology outpatients department for a follow‐up visit, during which the study catheter will be removed.

Finally, after the follow‐up visit, a patient subgroup will take part in a semi‐structured interview to evaluate their acceptability of the device used as well as their experiences, preferences and needs (at which point their contribution to the research will finish).

Health professionals who have participated in the study will complete at closure and after signing the informed consent form, a survey where they will evaluate their level of satisfaction and workload perceived for both types of catheters included in the study.

#### 5.2.1 Criteria for discontinuing or modifying allocated interventions

Reasons for going off or discontinuing the trial are as follows:

- Adverse events that may compromise the patient's safety.
- Need for antibiotic treatment for a reason unrelated to the use of a bladder catheter.
- The patient's refusal to continue (in the event that, according to clinical practice, they still needed to carry an indwelling bladder catheter; the T‐Control catheter would be exchanged for the conventional Foley‐type catheter).
- Intercurrent death.

### 5.3 Sample size determination

Assuming an independent *t* test and a significance level of 5%, we can achieve at least 80% power with a sample size of 25 per group and a dropout rate of 5% if the true effect size of the difference is 0.83 or greater. Based on previous in vitro studies comparing the T‐Control catheter versus the Foley‐type catheter, a large effect size is reasonable to anticipate. This sample size is also sufficient for comparing the rate of infections, which is one of the secondary endpoints of the study. Assuming a daily infection probability of 0.05^27^ for the control group and 0.015 for the intervention group, with an incremental risk of infection of 6.5% per day for both groups (estimated at around 3% to 10%),^13^, ^27^ we calculated a cumulative risk of 68% at the end of 14 days for the control group and 28% for the intervention group. To achieve 80% power and a significance level of 5%, we would need 23.5 patients in each group. Accounting for a possible 5% sample loss, a total of 50 patients will be recruited (25 per group).

### 5.4 Methods of data collection

The source and timing of measures are summarised in Table 1. In accordance with the proposed research objectives, the outcome measures that will be performed are the following.

**TABLE 1:** Source and timing of variables obtained for the study.

Variable	Method of obtention	Data collection format	Source	Timing			
				Baseline	2 weeks	Up 2 weeks AFUV	Close‐out
Baseline data (sociodemographic, clinical and life habits)	Personal interview/PCDN	Paper/electronic	P & HP	X			
Magnitude of infection	Urine culture/PCDN	Paper/electronic	Microbiology laboratory	X	X		
Rate of infection (Asymptomatic and symptomatic UTI)	Urine culture/PCDN	Paper/electronic	Microbiology laboratory	X	X		
Adverse events during catheter insertion	Observation/PCDN	Paper/electronic	HP	X			
Adverse events during catheterisation time	ID/PCDN	Paper/electronic	P		X		
Catheter biofilm	Viable cells counting/PCDN	Paper/electronic	Microbiology laboratory		X		
Antibiotic treatments related to catheterisation	ID/PCDN	Paper/electronic	P & HP		X		
Healthcare utilisation	ID/PCDN	Paper/electronic	P & HP		X		
Quantitative data for QoL and catheter satisfaction	Patients questionnaire & Equation 5D‐5L	Paper/electronic	P		X		
Qualitative findings for acceptability and experience	In‐depth interview	Audio recording	P			X	
Catheter opinion (professionals)	Professional questionnaire	Paper/electronic	HP				X
Workload perceived	NASA‐TLX questionnaire	Paper/electronic	HP				X

Abbreviations: AFUV, after follow‐up visit; EuroQoL 5D, EuroQol questionnaire – 5 dimensions – 5 levels; HP, health professional; ID, incident diary; NASA‐TLX, NASA Task Load Index questionnaire; P, participant/relative, friend or informal carer‐completed questionnaire; PCDN, Patient Clinical Data Notebook; QoL, Quality of Life perceived; UTI, urinary tract infections.

#### 5.4.1 Primary Outcome

##### Magnitude of infections

The magnitude of infection will be obtained from the analysis of urine culture samples taken from patients twice, during inclusion (time 0) and during withdrawal of the catheter (time 2 weeks; Table 1). In the event that the patient is prescribed an antibiotic for a UTI before the 14th day, the magnitude of infection will be evaluated at the time of prescription, through the analysis of a urine culture sample, according to usual clinical practice. The research staff will take between 5 and 10 ml of urine sample in a sterile container, which will be transported to the laboratory in the shortest time possible (<2 h). If the transport or processing cannot be performed immediately, the sample will be refrigerated between 2°C and 8°C without exceeding 24 h until processing. In the event that at the follow‐up visit the patient still needs to be catheterised, the urine sample will be collected from the new catheter inserted. Otherwise, the urine sample will be collected by the patient's spontaneous urination. After seeding the urine in specific culture media, the microorganisms that have grown in the culture will be analysed to determine the number and type of microorganisms present using techniques based on biochemical tests and MALDI‐TOF‐type mass spectrometry.

#### 5.4.2 Secondary outcomes

##### Rate of symptomatic and asymptomatic infections

The magnitude of infection obtained from the urine samples will be classified according to the following criteria:

- **Symptomatic infection**: The presence of pathogenic microorganisms in amounts greater or equal to 1000 CFU/ml accompanied with symptoms will determine the existence of the infection.
- **Asymptomatic infection**: In the absence of infection symptoms, a quantity of microorganisms greater than or equal to 100 000 CFU/ml will indicate asymptomatic infection.

In addition to the presence or absence of infections to assess their rate, this variable will also be collected using the outcome measure ‘infection‐free days’ (with a maximum possible value of 14 days) to be compared between Foley‐type catheters and T‐Control. Suppose the patient at some point prior to the follow‐up visit presents symptoms compatible with UTI. In that case, a urine culture will be performed to confirm the presence or absence of urinary infection, according to usual clinical practice. In the event that the patient receives an antibiotic for a UTI while he takes part in the study prior to the follow‐up visit, this will be recorded in the medical record. However, the patient will also be asked about it at the follow‐up visit when he hands in his incident diary, and this will be counted as an infection.

##### Self‐perceived HRQoL

The following instruments are administered to patients:

- **EuroQol‐5D‐5L (EQ‐5D‐5L)** ^28^: This is a generic HRQoL questionnaire that evaluates five domains: mobility, self‐care, usual activity, pain/discomfort and anxiety/depression. Each domain is scored on a 5‐point scale, which yields a descriptive system that can be combined into a five‐digit number reporting the patient's state of health. Each EQ‐5D‐5L health state can be converted to a single summary index by applying a formula that attaches weights to each level in each dimension. A number of formulae or value sets are available for different countries, based on the valuation of EQ‐5D health states from general population samples. In this study, the value set estimated for Spain by Ramos‐Goñi et al.^29^ will be used. After applying these weights, the range of the summary index is 1 (*perfect health*) to 0 (*health state equivalent to death*). Negative values represent health states considered to be worse than death. The questionnaire also includes a visual analogue scale (EQ‐VAS) where responders are asked to indicate their health status on the day of the interview, ranging from 0 (*worst possible health*) to 100 (*best possible health*).
- **Catheter‐related QoL questionnaire**: To evaluate the specific HRQoL for patients with AUR, Rethink Medical has developed a specific instrument created in the context of this project based on the experience of our previous studies (Patient's workshop).^25^ The questionnaire consists of 29 questions, related to the patient's experience and journey. These questions are related to the emotions felt at the beginning (right from the moment of the prescription), and during the catheter's usage, the acceptability and usability of the catheter itself and the accessories required (collection bag, plugs or others), personal satisfaction, adverse events and changes in the personal habits caused by the catheter's use. In order to quantitatively evaluate the answers, statements will include multiple answer options, single answer options and answer options with scores on a scale from 1 to 10. The questionnaire devised by Rethink Medical is expected to be validated during the different clinical studies scheduled for 2023 (RM‐TCONTROL‐2022‐01, RM‐TCONTROL‐2022‐02 and RM‐TCONTROL‐2022‐04).

##### Indication of antibiotic treatments

Indication of antibiotic treatments associated with catheter use. At the end of the follow‐up visit, antibiotic treatments will be recorded along with the dose and treatment time.

##### Determination of the biofilm

Determination of the biofilm formed in the catheters and identification of the microorganisms present. The catheter removed will be sent to the laboratory, and with the help of sterile gloves and scissors a 1 cm‐sized fragment corresponding to the part below, the balloon will be cultured for each catheter to assess whether biofilm is present or absent. The microorganisms forming the biofilm will be quantified as CFU/catheter piece. These microorganisms will also be identified by means of MALDI‐TOF‐type mass spectrometry to establish statistical relationships with those identified in the urine cultures. In the event that during the course of the 14 days in which the patient will be catheterised, he receives antibiotic treatment due to a UTI, this will be taken into account when analysing the results obtained in the catheter's biofilm determination.

##### Number of adverse events related to catheterisation

The type and number of adverse events will be registered in the Patient's Clinical Data Notebook: accidental disconnection of the catheter, obstruction, pain, loss of urine per catheter, haematuria and accidental spills caused by the professional during insertion. The patient will receive a Patient Incident Diary to write down the incidents, along with other information that may be of interest during catheterisation. Timing and source of adverse events data are summarised in Table 1.

##### Healthcare resource use

The costs arising from catheterisation, such as the consumption of consumable materials and resources, diagnostic tests (urine cultures and catheter cultures and biofilm analysis) will be collected in the Patient's Clinical Data Notebook and will be evaluated from the public healthcare services perspective. The analysis will also include costs because of patient contacts with primary care services, hospital admissions and length of stay, outpatient visits, emergency attendance and medications prescribed during the study period. The information related to healthcare resource use will be drawn from each patient's electronic clinical record (ECR).

##### Level of satisfaction and workload of health professionals

The following instruments are administered to health professionals:

- **NASA‐TLX** ^30^: This is a subjective, multidimensional and widely used evaluation tool that qualifies the perceived workload to evaluate the effectiveness of a task, system, equipment or other performance aspects. The questionnaire evaluates six dimensions (mental, physical and temporal demand, performance, effort and frustration), which enables rating them on a 1 to 10 scale, 1 being the lowest score and 10 the highest score.
- **Health professional satisfaction questionnaire**: The questionnaire specifically devised and based on the experience of previous LivingLab studies^24^ to quantitatively measure satisfaction with the devices used by health professionals (conventional Foley and T‐Control catheter). This questionnaire includes an initial section with 12 statements regarding the catheter insertion process. Health professionals will rate these statements for both devices used during the clinical trial on a 1 to 5 scale according to whether or not they agree with the statements. The second section is intended for health professionals to make a comparison between both devices by means of 11 statements for which they will have to indicate which device best fits these statements according to their opinion. They can only choose one device for each statement. Finally, the questionnaire consists of a free section in which health professionals can write any comments or suggestions. The usability of the device by professionals will also be also tested, verifying that the packaging is adequate and the instructions are understandable and contain all the information necessary to use the device safely.

##### Acceptability of the T‐Control device as well as the patient experience

Acceptability of the T‐Control device as well as the patient experience framed within the course of the disease: identification of the preferences and needs for training and information for use of the device and possible future improvements for the T‐Control device. A patient subgroup will be selected by means of an intentional theoretical sampling that seeks maximum variability in terms of the type of catheter inserted, age and the existence of infection. This selection will be made until information saturation is attained, which is estimated between 10 and 20 patients. For data collection, the in‐depth interview technique will be used with a semi‐structured script. Interviews will be audio recorded and transcribed. A thematic content analysis will be performed by processing subject responses with the qualitative software NVivo 12 (QSR International, Burlington, United States)^31^ and based on data preparation, identification of emerging problems, coding, interpretation, relativisation and determination of methodological rigour.^32^ Two independent researchers will code the data. The criteria of credibility, transferability, dependency and confirmability will be applied.

Baseline data for subjects such as sociodemographic (age, sex, education level, marital status, coexistence and type of health system user) as well as clinical (diagnostic, symptoms and previous use of bladder catheter and its accessories) and risk factors and toxic habits data (smoking habits, alcohol consumption and sedentary lifestyle).

### 5.5 Data management

The data will be stored in a secure computer database and kept confidential in accordance with Europe Union and Spanish Data Protection Legislation (General Data Protection Regulation and the Data Protection Act 2018). Personal data are not kept longer than necessary for the purpose for which it is processed. Access rights to the data set are managed. The principal investigators of each centre and the research coordinator will have access to the full dataset to enable analysis at the trial end, while the trial statistician, who is part of the CRO, will only have access to the participants' code. Authorised representatives or Competent Authorities will gain access to those portions of the medical records relevant to the clinical research by means of cross‐reference with clinical research personnel to verify the data if required. All computerised data will be identified solely by a code. All essential data and documents will be kept for a period of at least 10 years after the trial ends.

The personnel in charge of monitoring will enter the data collected by the research team at the centres into the study database. Safety data, Case Report Forms (CRFs) and subject questionnaires, as well as biological sample results, will be entered into the database. Monitoring staff will work closely with the research teams at the centres to ensure that the data are as complete and accurate as possible. Data quality is improved by means of a wide range and consistency checks included in the monitoring activities.

The semi‐structured interviews will be performed by researchers experienced in qualitative research. The data will be added to the database by the staff in charge of monitoring.

### 5.6 Oversight and monitoring

An independent CRO will be in charge of monitoring the clinical trial. This CRO includes staff with clinical, statistical and methodological expertise. The Sponsor will meet with the CRO after every monitoring visit at the centres to ensure the trial is executed and carried out properly, make recommendations and report any event that has to be passed on to the Ethics Committee or the competent regulatory health authorities.

There are no scheduled interim analyses for efficacy or futility. However, during the clinical study, the external monitor will have regular contact with the centres where the study is performed. These contacts will include visits to confirm that the facility is in accordance with specified standards and that the clinical research teams are performing the procedure as directed, as well as trial progress and any safety issues.

Retention of subjects is promoted by regular contact with the staff responsible for the study and ensuring adequate outcome measures collection. All data collected are retained and used in the analysis. Data from subjects who terminate the study earlier will also be included in a substudy analysis.

Deviations from the allocated study will be recorded in the CRFs and evaluated as a secondary endpoint.

### 5.7 Analysis plan

A statistical analysis plan will document the scheduled analysis, to be finalised before the data lock. The final analysis will take place after full recruitment and follow‐up, at the end of the study.

Demographic, clinical factors and toxic habits will be summarised using the appropriate descriptive statistics and graphical summaries according to the type of catheter. All continuous variables will be summarised using the following descriptive statistics: n, mean, standard deviation, 95% confidence interval (95% CI), maximum, minimum, median and interquartile range, and all categorical variables as counts and percentages.

The main study variable (the magnitude of infections due to catheter use) will be compared by type of catheter using the Student *t* test for independent samples or the Mann–Whitney U test (in case of non‐normality). In addition, the percentage of patients with UTI due to catheter use will be compared by the type of catheter using Fisher exact test for independent proportions. For the secondary analysis, continuous variables will be analysed using the parametric Student *t* test for independent samples or the Mann–Whitney U test in case of non‐normality. Normality will be analysed with the Shapiro–Wilk test and Q‐Q plot exploration. Two‐tailed *P* < 0.05 will be considered statistically significant. R Statistical Software (v4.2.1; R Core Team 2021)^33^ will be used for all analyses.

Missing data will be reported. If required, multiple imputation methods will be used for missing endpoint data.

#### 5.7.1 Subgroup analyses

A subgroup analysis is scheduled for age groups (50–65 vs. >65 years) with a 95% statistical significance level. An interaction effect between age and type of catheter will be evaluated. Patients who do not complete the study will also comprise a subgroup, and data will be analysed separately from the patient subgroup who complete the study from those who do not. These analyses will be exploratory since we will not have sufficient power for interaction analysis.

#### 5.7.2 Cost‐effectiveness analysis

An economic evaluation of T‐Control versus the conventional Foley catheter from the public healthcare services perspective will be performed according to the analytical methods accepted by the scientific community.^34^ The cost‐effectiveness measure will be the incremental cost per quality‐adjusted life year (QALY) gained. QALYs are a generic health measure that combines information on life expectancy with the patient's quality of life. QALYs will be calculated based on the HRQoL data collected according to the EQ‐5D‐5L instrument, which will be collected for each patient. The time horizon will be the study duration. Costs included in the analysis will be those incurred by the public healthcare service. Unit costs will be obtained from the hospital centres accounting records whenever possible, from the eHealth cost database (Oblikue Consulting) and from national public sources. The mean total cost of each intervention evaluated will be presented using basic descriptive statistics (means, medians and measures of variability such as variance).

Cost‐effectiveness will be calculated as the incremental cost‐effectiveness ratio (ICER), which results from dividing the difference in costs between interventions by the difference in effects observed (QALYs). Nonparametric methods based on bootstrapped simulations will be used to calculate the confidence intervals around the ICER. In addition, the same nonparametric methods will be used to construct a cost‐effectiveness acceptability curve that will reveal the probability that each alternative is cost‐effective for different cost‐effectiveness thresholds (willingness to pay for an additional unit of effectiveness). Finally, deterministic sensitivity analysis (one‐, two‐ and multi‐way) will be performed with the aim of analysing the impact of the parameters on the cost‐effectiveness results. The analysis will be performed using the software R and the statistical package STATA (StataCorp LLC, College Station, United States).

## 6 DISCUSSION

This is the very first clinical trial to investigate the new T‐Control device. It is important to note that one of the limitations of the study is although the results analysis team will be blinded by a unique code given to each patient, neither the participants nor the health professionals, the research team involved and the monitoring team the research team involved and the monitoring team will be blinded. However, the use in the study of both objective and subjective tools to evaluate the different aspects of bladder catheterisation related to patients (infections, adverse events, biofilm formation, quality of life and acceptance of the device), health professionals (burden work and device satisfaction) and impact on the health system (antibiotic treatment prescription and associated costs) is a strength that also should be remarked.

In this way, the study could contribute to improving health (infection prevention) and social well‐being (greater quality of life, autonomy and ability to live with the disease) among patients who use permanent bladder catheters, although it is true that the patients taking part in the study will only be men with acute urine retention, which on the one hand enables a more homogeneous sample but on the other means that the results cannot be extrapolated to other cohorts or conditions for the use of T‐Control.

It also aims to promote an active and healthy lifestyle, preventing negative consequences for users. From the point of view of the work environment, the project tries to improve the working conditions of health personnel, reducing occupational risks such as spillage and contamination by contact with urine and offering an easy‐to‐use product that could make it easier to comply with bladder catheter insertion protocols without difficulty or need for additional help.

The development of this clinical study could help the sustainability of the Health System, since it offers a profitable product, with the potential to reduce infections, emergency visits (including the possible need for hospitalisation), personnel costs and so forth. Reducing the need for emergency healthcare visits, with or without admission to hospital care, is of particular interest as the Spanish healthcare system is under severe pressure, partly due to the existing shortage of registered nurses. It also aims to contribute to improving public health conditions, reducing the use of antibiotics and mitigating the risks of transmission during pandemics such as COVID‐19.

## AUTHOR CONTRIBUTIONS

All the authors have contributed to the study design process and manuscript writing. Ana Toledo Chávarri and Lilisbeth Perestelo‐Pérez are responsible for the design of the subgroup qualitative analysis of the acceptability of the new T‐Control device and patient experience. Cristina Valcárcel‐Nazco and Lidia García‐Pérez are responsible for the design of cost‐effectiveness analysis and economic evaluation. PSA and ML are responsible for the conception of the study and its coordination. Manuel Luque González, Marta Serrano Muñoz, Clara Armas Moreno and Max Mòdol Vidal are responsible for the design of the catheter's biofilm analysis protocol. Laura Vera Álamo and Adrián Amador Robayna are the principal clinical investigators, Pedro Raúl Castellano Santana is responsible for the clinical coordination between centres and Yolanda Ramallo‐Fariña and Miguel Ángel García‐Bello are responsible for the methodological design of the clinical trial and statistical analysis plan. Yolanda Ramallo‐Fariña and Max Mòdol Vidal drew up the initial manuscript draft. All authors made substantial contributions to the revising of the manuscript and approved the final version.

## CONFLICT OF INTEREST

This group of authors (YRF, ATC, CVN, LPP, LGP, MAGB and PSA) belongs to the Evaluation Unit (SESCS), Canary Island Health Service (SCS) (www.sescs.es), an institution of the Canary Islands Government that is also a member of the Spanish Network for Research on Chronicity, Primary Care and Health Promotion (RICAPPS, www.ricapps.es). This article is part of the early dialogues activity carried out by the SESCS as an Evaluation agency and declares that they have no competing interests. ML, MS, CA and MM are part of Rethink Medical S.L., which is the sponsor of this study, owning the rights of T‐Control. LV, AA and PRC declare that they have no competing interests.

## Supporting information


**Data S1.** Supplementary Material.

**Data S2.** Supplementary Material.

**Data S3.** Supplementary Material.

**Data S4.** Supplementary Material.

**Data S5.** Supplementary Material.
