# Supplementary material for: Effectiveness of the T‐Control catheter: A study protocol
Source: BJUI Compass. 2023 Dec 4;5(2):178–88. doi: 10.1002/bco2.285 (PMC10869656; doi:10.1002/bco2.285)

**Instructions for use of the Silicone T-Control® Foley Catheter**

**Product data**

**Product or trade Name:** T-Control®

**Manufacturer:** Rethink Medical S.L.

**Model and sizes: Two-way urethral silicone catheter of 14FR, 16FR and, 18FR sizes.**

**Description**

T-Control® is a Foley 2-way silicone urethral catheter with an integrated fluid control valve for indwelling drainage, made of silicone, latex-free, sterile and single-use.

Class IIa product. Sterile by Ethylene Oxide (EO). The sterility of the product is only ensured as long as the package is not opened or damaged and not beyond the expiration date.

The continuous use of a single device of T-Control® cannot exceed 30 days. The cumulative use for each type of T-Control® device can exceed 30 days.

**Presentation**

Unit of use: Peelable sealed container.

**Intended user**

Healthcare professionals.

Intended patient population: Adult men and women.

**Intended purpose / Indications**

**T-Control®** is a tubular device intended for being introduced into the bladder cavity through the urethra of the patient. It is fixed in the bladder by filling the anchoring balloon to drain urine by intravesical pressure.

The use of T-Control® is indicated for different dysfunctions such as urinary retention, incontinence with wounds in the sacrum or in areas that may be in contact with urine and make it difficult to heal, control of urine production, palliative care, surgery and other functions such as residual volume measurement or obtaining a urine sample.

**Instructions for use**

Please read the following instructions before using T-Control®:

| **Step 1**  Before catheter insertion, the patient's urethral meatus or vulva should be cleaned and disinfected according to your unit's protocol (fig.1). | 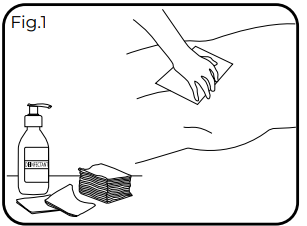 |
| --- | --- |
| **Step 2**  Wearing sterile gloves and always following the appropriate asepsis measures, open the sterile package of the catheter (Fig.2), take the catheter and use a syringe (without a needle) to check if the balloon can be filled or emptied (Fig.3) with the maximum volume of distilled water, indicated on the catheter and the bag label.  **Warning**: Do not change the position of the catheter valve yet, because it is in the correct position for the INSERTION process. | 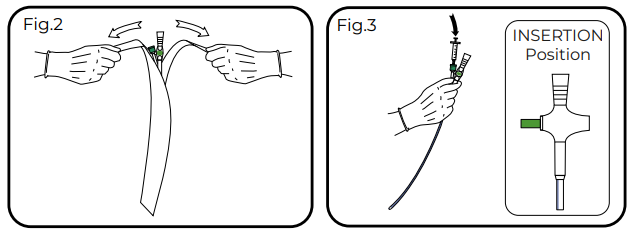 |
| **Step 3**  Lubricate the catheter according to current health protocol. Among the options, we highlight the lubrication of 3-4 cm from the tip of the catheter (Fig.4) or the lubrication through the patient's urethral meatus (Fig.5). | 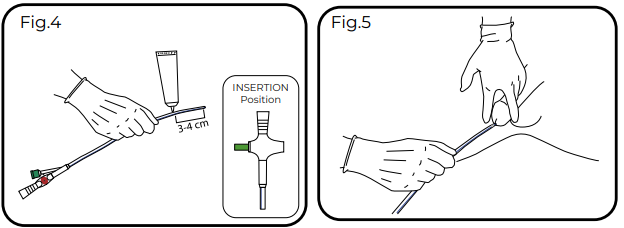 |
| **Step 4**  Insert the catheter into the bladder approximately 3 - 4 cm to place the balloon completely in the bladder (Fig.6). Generally, resistance will be noticed when reaching the bladder and once the catheter is inserted inside, the urine will begin to flow through the tube of the catheter if the bladder is not empty (Fig.7). The urine will flow towards the valve in an interval between 5 and 20 seconds, depending on the amount of urine accumulated in the bladder. The urine will not come out. In case the urine takes more than 20 seconds to flow, put the valve in OPEN position. | 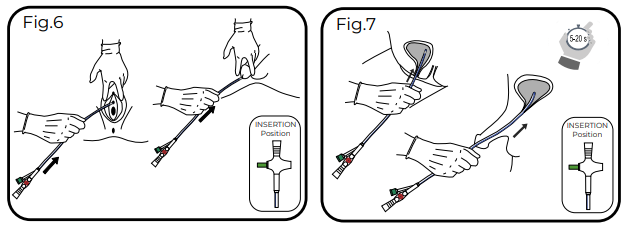 |
| **Step 5**  Once the descent of urine is visually perceived, the balloon can be inflated with 10cc of sterile water using a syringe (Fig.8). | 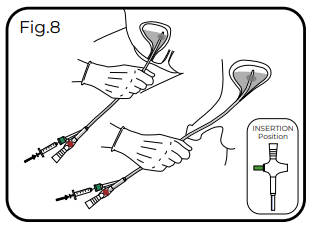 |
| **Step 6**  Gently pull on the catheter to check that the balloon is attached to the bladder (Fig.9). | 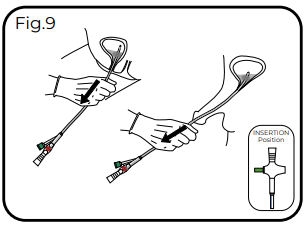 |
| **Step 7**  Once the balloon is filled, operate the valve preferably with three fingers (as indicated in the image) until it passes from the INSERTION to the CLOSED position, the red colour remaining visible and the white safety cap at the other end of the green part coming off of the valve (Fig.10).  **Warning**: This cap has no use once the device has been activated for the first time, so it has to be discarded. | 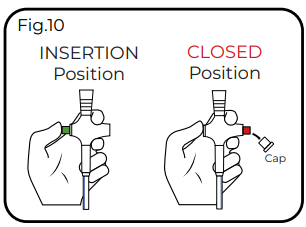 |

**Urine Sampling**

| To collect the sample, place a collection container on a flat surface without inserting the catheter cone inside the container or letting it touch its wall (Fig.11), move the valve to the OPEN position, leaving the green colour visible (Fig.12).  After taking the sample, move the valve back to the CLOSED position (Fig.13). | 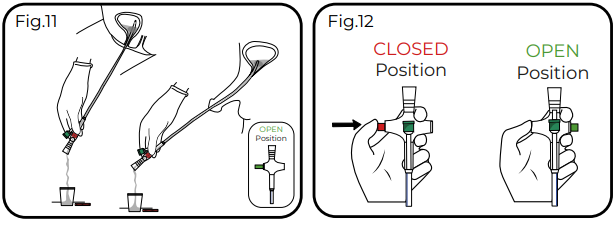  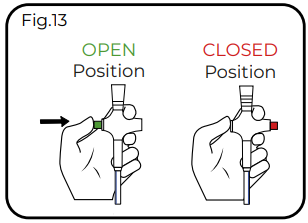 |
| --- | --- |

**Empty the bladder with T-Control®**

| **We recommend washing hands with soap before handling the catheter.**  **Step 1**  To empty the bladder, if necessary, while facing the urinal or sitting down, move T-Control® to the OPEN position (Fig.26 and Fig.27). | 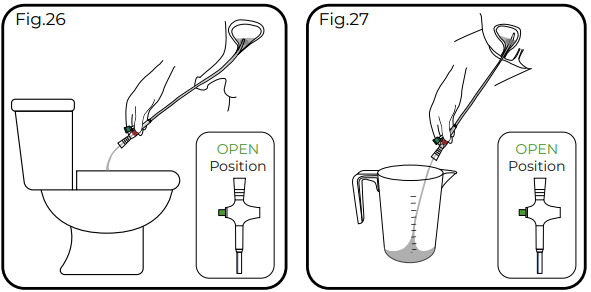 |
| --- | --- |
| **Step 2**  When the urine stops flowing, move T-Control® to the CLOSED position (Fig.28 and Fig.29).  Clean the catheter cone to prevent urine stains on clothing. | 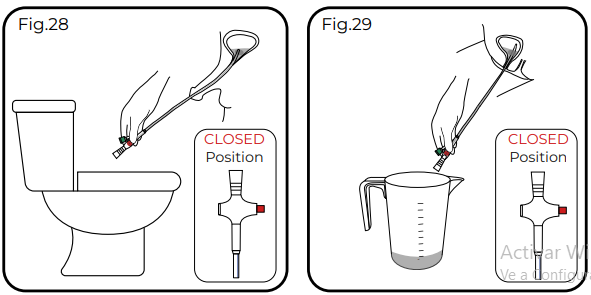 |

**Use of collection bag**

| **Step 1**  Before placing the urine collection bag, check that the valve is in the CLOSED position to prevent urine leakage (Fig.14). | 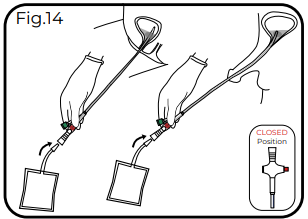 |
| --- | --- |
| **Step 2**  Connect the collection bag (Fig.14) and turn the valve to the OPEN position (Fig.15). The urine will begin to fill the collection bag (Fig.16). | 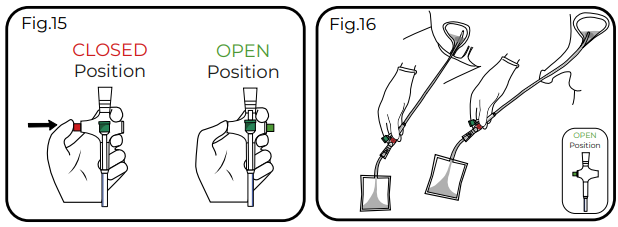 |
| **Step 3**  If you want to interrupt the continuous drainage to the collection bag to allow the bladder to fill or to prevent possible accidental leaks during the transfer or mobilisation of the patient, you can activate the CLOSED position again (Fig.17). Urine will stop flowing into the collection bag (Fig.18). | 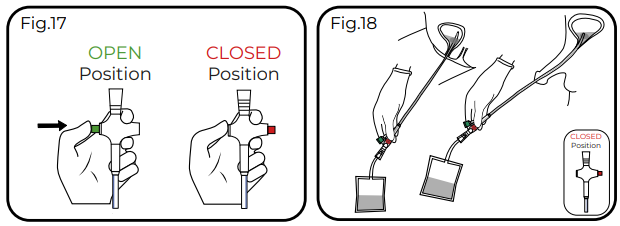 |

**T-Control® attach**

**The attach of the T-Control® catheter can be done:**

| Directly connected T-Control® to the bag previously attached to the leg. With T-Control® in the CLOSED position (Fig.19) the flow of urine to the bag is blocked. When T-Control® goes to the OPEN position (Fig.20) the urine begins to fill the bag. | 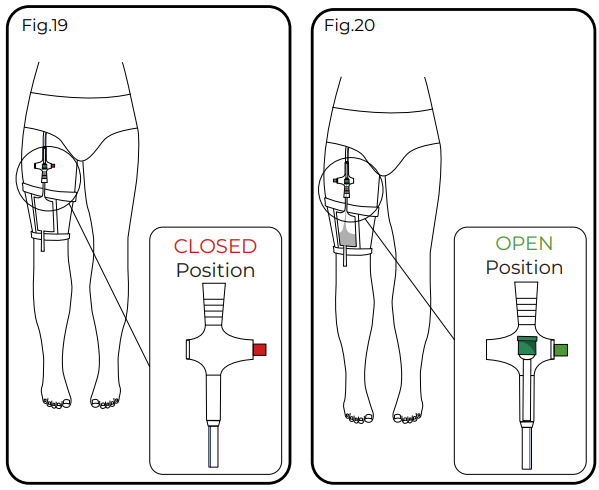 |
| --- | --- |
| Using the Holder, with the T-Control® in the CLOSED position, it connects to the Holder (Fig.21), and then you can choose to use a thigh strap as shown in figures (Fig.22) and (Fig. 23) or by directly attaching the Holder to underwear, as shown in figures (Fig.24) and (Fig.25). | 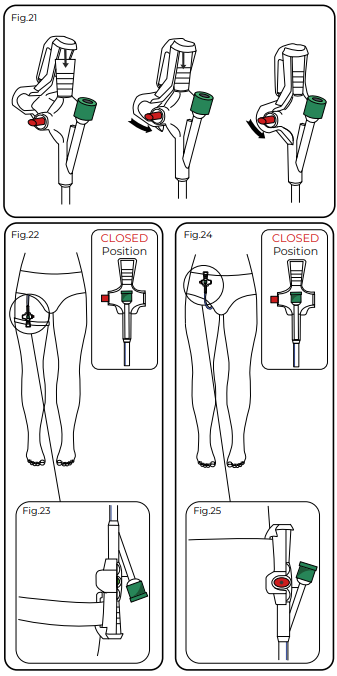 |

Catheter removal

Before removing the catheter, empty the bladder, make sure the valve is in CLOSED position and deflate the balloon by aspirating the water from it with a needle-free syringe. Then, gently remove the catheter. Optionally, in case of observing any deflation error or in case of needing rapid deflation of the balloon, you can cut the catheter shaft below the inflation valve to then remove the catheter.

**Contraindications**

1. Urinary tract infection (cystitis/urethritis).
2. Injury to the urethra.
3. Urethral stricture.
4. Injury to the penis.
5. Insufficient mental and/or physical capacities to handle the device
6. Ureteral reflux.
7. Overactive bladder.
8. Bilateral obstructive uropathy

**Side effects**

Side effects associated with T-Control® are the same as those associated with Foley-type catheters: septicaemia, catheter-associated urinary tract infections (CAUTI) and biofilm proliferation, urethral damage, injury to the bladder, meatus or urethral erosion, narrowing of the urethra, bladder spasms, etc.

If any discomfort or indication of trauma or infection occurs, consult your doctor. Any serious incident that occurs while using the bladder catheter should be reported to the manufacturer and local health authorities.

Prec**autions**

1. The absence of leaks and the correct filling of the balloon must be checked before use.
2. Make sure the syringe is properly inserted into the fill valve hole.
3. Check the capacity of the balloon on the individual outer packaging.
4. Do not overfill or underfill the balloon or the catheter could come off.
5. The balloon can break if it is filled beyond its capacity, impacted by sharp objects or by crystals (such as those produced by physiological serum). Periodically check if the balloon is well anchored, and follow the recommendations of the current health protocol.
6. Make sure that the white safety cap, located at the other end of the valve to the green button, is in place before using the catheter and that it comes off when the device is activated. Be careful when activating the device for the first time since the cap can be projected in the direction in which it is pointed.
7. Change the catheter in case the valve is blocked.
8. Avoid storing the product together with chemicals.

**Warnings**

1. For single-patient use only.
2. Do not use it if the package has been opened or damaged.
3. Please use the product immediately after opening the package.
4. The product is intended to be inserted by competent healthcare professionals and aseptic technique should be used.
5. Do not remove the catheter by yourself. This must be removed by a competent professional.
6. Do not use petroleum-based ointments or lubricants, it may damage the catheter.
7. Do not use the product if the balloon is broken.
8. Fill the catheter balloon with sterile distilled water. Do not use needles to fill the balloon.
9. Do not clamp the catheter body, as this may damage the catheter and prevent balloon emptying.
10. Do not use it after the expiration date.
11. This is a single-use disposable product, any re-sterilization or reuse of the product may pose a danger to the patient.
12. Do not use the catheter without restraint during moderate or intense activity.
13. Keep out of reach of pets.
14. Dispose of the catheter and its packaging in accordance with the national regulations in force.
15. The proper functioning of the device is not guaranteed if it is not used according to Fig. 10, 12, 13, 15 and 17.

**Conditions of conservation**

1. Store in its container in a cool, dry place, protecting the product from humidity and excessive heat.
2. Avoid prolonged exposure to light.
3. Store in such a way that the product is not crushed.
4. In case of observing visible sediment in the system, follow the recommendations of the healthcare professional.


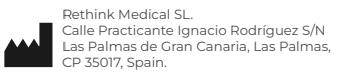


In case of doubt contact: info@rethinkmedical.es

**Meaning of the symbols on the packaging:**


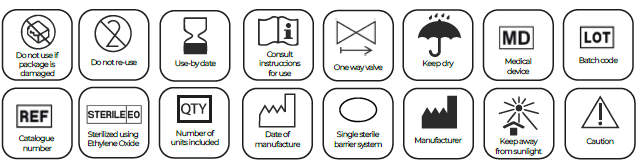

Supplement: Supplementary file 1 — Data S1. Supplementary Material. [file BCO2-5-178-s005.docx]
