# Supplementary material for: Effectiveness of the T‐Control catheter: A study protocol
Source: BJUI Compass. 2023 Dec 4;5(2):178–88. doi: 10.1002/bco2.285 (PMC10869656; doi:10.1002/bco2.285)
Supplement: Supplementary file 2 — Data S2. Supplementary Material. [file BCO2-5-178-s002.docx]

**Instructions for use of the Silicone T-Control® Foley Catheter**

**Product or trade name:** T-Control®

**Manufacturer:** Rethink Medical S.L.

**Description**

T-Control® is a Foley 2-way silicone urethral catheter with an integrated fluid control valve for indwelling drainage, made of silicone, latex-free, sterile and single-use.

Class IIa product. Sterile by Ethylene Oxide (EO). The sterility of the product is only ensured as long as the package is not opened or damaged and not beyond the expiration date.

The continuous use of a single device of T-Control® cannot exceed 30 days. The cumulative use for each type of T-Control® device can exceed 30 days.

**Intended purpose / Indications**

**T-Control®** is a tubular device intended for being introduced into the bladder cavity through the urethra of the patient. It is fixed in the bladder by filling the anchoring balloon to drain urine by intravesical pressure.

The use of T-Control® is indicated for different dysfunctions such as urinary retention, incontinence with wounds in the sacrum or in areas that may be in contact with urine and make it difficult to heal, control of urine production, palliative care, surgery and for other functions such as residual volume measurement or obtaining a urine sample.

**T-Control® must be inserted by qualified healthcare personnel.**

**Instructions for use after insertion**

Please read the following instructions before using T-Control®:

**Instructions on valve actuation**

| Operate preferably with three fingers (as indicated in the image) the valve until it passes from the INSERTION position to the CLOSED position, the red colour being visible (Fig.10). | 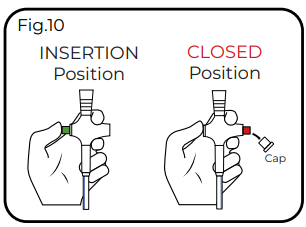 |
| --- | --- |

**Empty the bladder with T-Control**®

| **We recommend washing hands with soap before handling the catheter.**  **Step 1**  To empty the bladder, if necessary, while facing the urinal or sitting down, move T-Control® to the OPEN position (Fig.26 and Fig.27). | 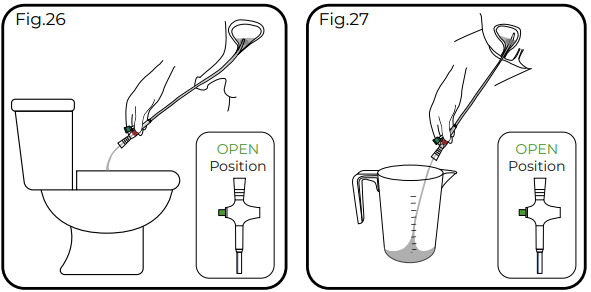 |
| --- | --- |
| **Step 2**  When the urine stops flowing, move T-Control® to the CLOSED position (Fig.28 and Fig.29).  Clean the catheter cone to prevent urine stains on clothing. | 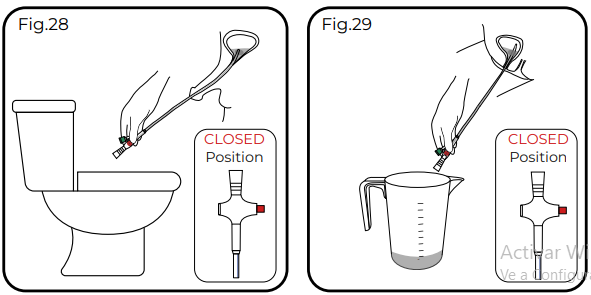 |

**Use of collection bag**

| **Step 1**  Before placing the urine collection bag, check that the valve is in the CLOSED position to prevent urine leakage (Fig.14). | 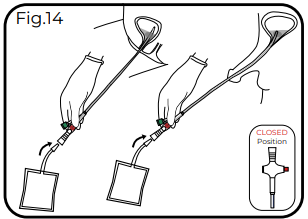 |
| --- | --- |
| **Step 2**  Connect the collection bag (Fig.14) and turn the valve to the OPEN position (Fig.15). The urine will begin to fill the collection bag (Fig.16). | 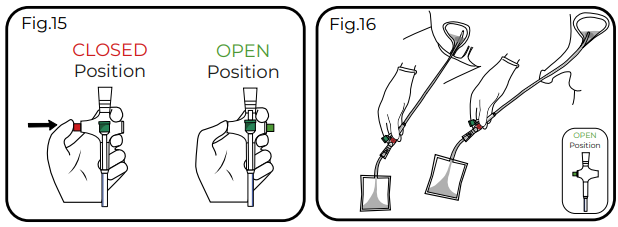 |
| **Step 3**  If you want to interrupt the continuous drainage to the collection bag to allow the bladder to fill or to prevent possible accidental leaks during the transfer or mobilisation of the patient, you can activate the CLOSED position again (Fig.17). Urine will stop flowing into the collection bag (Fig.18). | 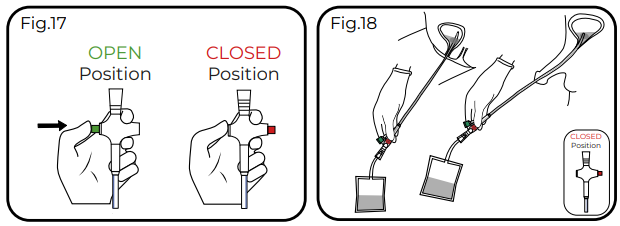 |

**T-Control**® **attach**

**The attach of the T-Control® catheter can be done:**

| Directly connected T-Control® to the bag previously attached to the leg. With T-Control® in the CLOSED position (Fig.19) the flow of urine to the bag is blocked. When T-Control® goes to the OPEN position (Fig.20) the urine begins to fill the bag. | 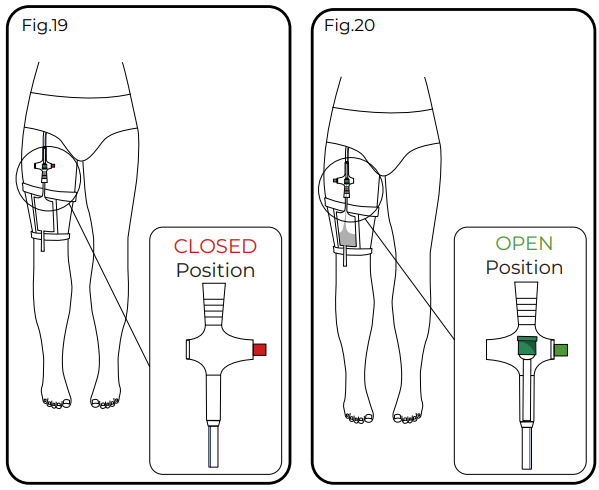 |
| --- | --- |
| Using the Holder, with the T-Control® in the CLOSED position, it connects to the Holder (Fig.21), and then you can choose to use a thigh strap as shown in figures (Fig.22) and (Fig. 23) or by directly attaching the Holder to underwear, as shown in figures (Fig.24) and (Fig.25). | 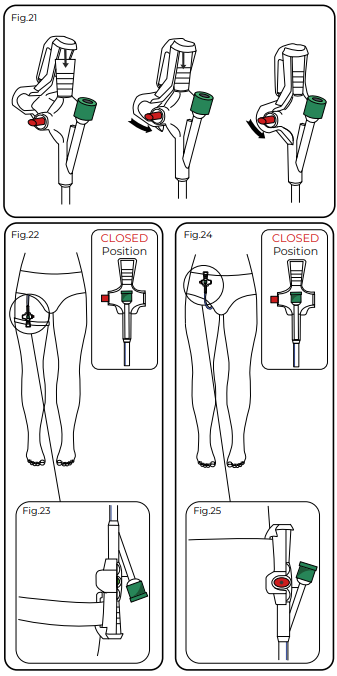 |

**Contraindications**

1. Urinary tract infection (cystitis/urethritis).
2. Injury to the urethra.
3. Urethral stricture.
4. Injury to the penis.
5. Insufficient mental and/or physical capacities to handle the device.
6. Ureteral reflux or kidney failure.
7. Overactive bladder.
8. Bilateral obstructive uropathy.

**Side effects**

Side effects associated with T-Control® are the same as those associated with Foley-type catheters: septicaemia, catheter-associated urinary tract infections (CAUTI) and biofilm proliferation, urethral damage, injury to the bladder, meatus or urethral erosion, narrowing of the urethra, bladder spasms, etc.

If any discomfort or indication of trauma or infection occurs, consult your doctor. Any serious incident that occurs while using the catheter should be reported to the manufacturer and local health authorities.

**Warnings**

1. For single-patient use only.
2. The product is intended to be inserted by competent healthcare professionals and aseptic technique should be used.
3. Do not remove the catheter by yourself. This must be removed by a competent professional.
4. Do not use petroleum-based ointments or lubricants, it may damage the catheter.
5. Do not clamp the catheter body, as this may damage the catheter and prevent balloon emptying.
6. This is a single-use disposable product, any re-sterilization or reuse of the product may pose a danger to the patient.
7. Do not use the catheter without restraint during moderate or intense activity.
8. Keep out of reach of pets.
9. The proper functioning of the device is not guaranteed if it is not used according to Fig. 10, 12, 13, 15 and 17.
