# Supplementary material for: Effectiveness of the T‐Control catheter: A study protocol
Source: BJUI Compass. 2023 Dec 4;5(2):178–88. doi: 10.1002/bco2.285 (PMC10869656; doi:10.1002/bco2.285)
Supplement: Supplementary file 4 — Data S4. Supplementary Material. [file BCO2-5-178-s001.docx]

**START OF THE STUDY**

DATE: ………………………………… PATIENT CODE: …………………………..

**Have I been instructed after the catheterization in self-care?** YES NO

**Have I been instructed to fill in the patient or incident diary?**  YES NO

**INCIDENT DIARY**

Following the instructions indicated by the professionals who have given you this diary, you will write down in the Patient Incident Diary the information corresponding to any incident that you may have observed during your participation in this study, as well as any symptoms or discomfort that you may have experienced. You will need to bring the diary to the medical office on your next visit.

To complete it, you must follow the guidelines set out:

| **Incident (in case any of the mentioned incidents arise, write down the date and mark with an “X”)** | | | | | | | |
| --- | --- | --- | --- | --- | --- | --- | --- |
| **Date** | **Infections** | **Accidental disconnection** | | **Presence of blood in urine**  **(by catheter or in bag)** | | **Accidental leak** | |
|  |  |  | |  | |  | |
|  |  |  | |  | |  | |
|  |  |  | |  | |  | |
|  |  |  | |  | |  | |
|  |  |  | |  | |  | |

| **Incident (in case any of the mentioned incidents arise, write down the date and mark with an “X”)** | | | | | | | |
| --- | --- | --- | --- | --- | --- | --- | --- |
| **Date** | **Emergency care** | | **Catheter change** | | **Catheter clogging** | | **Pain/Traction** |
|  |  | |  | |  | |  |
|  |  | |  | |  | |  |
|  |  | |  | |  | |  |
|  |  | |  | |  | |  |
|  |  | |  | |  | |  |

| **Signs and Symptoms (if any, write down the date and mark with an “X”)** | | | | | | | |
| --- | --- | --- | --- | --- | --- | --- | --- |
| **Date** |  |  |  |  |  |  |  |
| Fever |  |  |  |  |  |  |  |
| Shaking chills |  |  |  |  |  |  |  |
| Hypotension |  |  |  |  |  |  |  |
| Tachycardia |  |  |  |  |  |  |  |
| Others  (specify) |  |  |  |  |  |  |  |
|  |  |  |  |  |  |  |  |

| **Antibiotics during the study** | | | | | **General discomfort** | | | |
| --- | --- | --- | --- | --- | --- | --- | --- | --- |
| Drug | Dose | Frequency | Start | End | From | To | From | To |
|  |  |  |  |  |  |  |  |  |
|  |  |  |  |  |  |  |  |  |
|  |  |  |  |  |  |  |  |  |
|  |  |  |  |  |  |  |  |  |
|  |  |  |  |  |  |  |  |  |

**REMARKS**

DATE: ……………………………..

…………………………………………………………………………………………………………

…………………………………………………………………………………………………………

…………………………………………………………………………………………………………

…………………………………………………………………………………………………………

DATE: ……………………………..

…………………………………………………………………………………………………………

…………………………………………………………………………………………………………

…………………………………………………………………………………………………………

…………………………………………………………………………………………………………

DATE: ……………………………..

…………………………………………………………………………………………………………

…………………………………………………………………………………………………………

…………………………………………………………………………………………………………

…………………………………………………………………………………………………………

DATE: ……………………………..

…………………………………………………………………………………………………………

…………………………………………………………………………………………………………

…………………………………………………………………………………………………………

…………………………………………………………………………………………………………

DATE: ……………………………..

…………………………………………………………………………………………………………

…………………………………………………………………………………………………………

…………………………………………………………………………………………………………

…………………………………………………………………………………………………………
